# Laser Capture Microdissection and RNA-Seq Analysis: High Sensitivity Approaches to Explain Histopathological Heterogeneity in Human Glioblastoma FFPE Archived Tissues

**DOI:** 10.3389/fonc.2019.00482

**Published:** 2019-06-07

**Authors:** Prospero Civita, Sara Franceschi, Paolo Aretini, Valerio Ortenzi, Michele Menicagli, Francesca Lessi, Francesco Pasqualetti, Antonio Giuseppe Naccarato, Chiara Maria Mazzanti

**Affiliations:** ^1^Fondazione Pisana per la Scienza ONLUS, Pisa, Italy; ^2^Department of Translational Research and New Technologies in Medicine and Surgery, Pisa University Hospital, Pisa, Italy; ^3^Radiation Oncology, Pisa University Hospital, Pisa, Italy

**Keywords:** glioblastoma, microenvironment, LCM, RNA-seq, tumor heterogeneity

## Abstract

Laser capture microdissection (LCM) coupled with RNA-seq is a powerful tool to identify genes that are differentially expressed in specific histological tumor subtypes. To better understand the role of single tumor cell populations in the complex heterogeneity of glioblastoma, we paired microdissection and NGS technology to study intra-tumoral differences into specific histological regions and cells of human GBM FFPE tumors. We here isolated astrocytes, neurons and endothelial cells in 6 different histological contexts: tumor core astrocytes, pseudopalisading astrocytes, perineuronal astrocytes in satellitosis, neurons with satellitosis, tumor blood vessels, and normal blood vessels. A customized protocol was developed for RNA amplification, library construction, and whole transcriptome analysis of each single portion. We first validated our protocol comparing the obtained RNA expression pattern with the gene expression levels of RNA-seq raw data experiments from the BioProject NCBI database, using Spearman's correlation coefficients calculation. We found a good concordance for pseudopalisading and tumor core astrocytes compartments (0.5 Spearman correlation) and a high concordance for perineuronal astrocytes, neurons, normal, and tumor endothelial cells compartments (0.7 Spearman correlation). Then, Principal Component Analysis and differential expression analysis were employed to find differences between tumor compartments and control tissue and between same cell types into distinct tumor contexts. Data consistent with the literature emerged, in which multiple therapeutic targets significant for glioblastoma (such as Integrins, Extracellular Matrix, transmembrane transport, and metabolic processes) play a fundamental role in the disease progression. Moreover, specific cellular processes have been associated with certain cellular subtypes within the tumor. Our results are promising and suggest a compelling method for studying glioblastoma heterogeneity in FFPE samples and its application in both prospective and retrospective studies.

## Introduction

Glioblastoma (GBM) is considered the most malignant primary tumor of the brain accounting for ~54% of all gliomas and 16% of all primary brain tumors (1). Although standard treatment at diagnosis is multimodal and include surgical resection, radiation, and systemic chemotherapy, patients usually have a median survival of ~14.6 mos. from diagnosis (2). The poor prognosis of GBM is mainly due to its diffuse infiltrative growth into the surrounding brain (3), making it extremely difficult to treat by total surgical resection or chemo-radiotherapy (4) and delaying the efficacy of treatments.

GBM is composed of an interactive network of neoplastic and non-neoplastic cells, such as microglia/macrophages, that account for about 40% of the tumor mass, reactive astrocytes, fibroblasts, pericytes and immune cells (5). These well-established networks, characterized by cell-cell interactions and connections of cellular compartments, create a complex micro-environment that constantly gives signals, activating cells migration and developing finally permissive niches that promote cancer cells survival and proliferation (6).

Histopathologic features, that distinguish GBM from lower grade astrocytomas, are found near the contrast-enhancing rim that surrounds the tumor core and includes (1) foci of necrosis, usually with evidence of surrounding cellular pseudopalisades (“pseudopalisading necrosis”); (2) microvascular hyperplasia, a form of angiogenesis morphologically recognized as endothelial proliferation within newly sprouted vessels (7), and (3) “perineuronal satellitosis,” typical grouping of neoplastic astrocytes around neurons when the tumor infiltrates the gray matter (8). Connections between these cells compartments play an important role in the development and malignant progression of glioblastoma.

Therefore, it is essential to study the expression profiles of each cell compartment independently, to clarify the interaction between cells and their microenvironment.

To this end, we have applied laser capture microdissection (LCM) to isolate groups of cells from specific tissue compartments of human GBM FFPE tissues. LCM is a cutting-edge technology for isolating pure cell populations from a heterogeneous tissue sample. It can accurately target and capture cells of interest for a wide range of downstream analyses (9). Several studies have questioned tumor heterogeneity using samples of fresh frozen tissues (FF) or biopsies, encountering some relative limitations such as low starting material and privileged diagnostic procedures, lack of clinical annotations or long-term follow-up (10). FFPE tissue samples stored in diagnostic pathology biobank represent a suitable material to overcome these limitations and becoming an attractive source for retrospective and prospective studies. Moreover, the process of formalin-fixed paraffin-embedding is so far the most suitable histological method for stabilizing and preserving tissues with native morphology and cellular structures. FFPE tissues can be stored long term at room temperature and used over decades. For these reasons, despite poor quality and quantity of nucleic acids extracted from FFPE tissue, the number of molecular studies incorporating FFPE material is increasing.

In the last decade the advances in next-generation sequencing (NGS) technology has allow to explore a wide range of molecular analysis (i.e., genomic, epigenomic, and transcriptomic) using limited quality/quantity of material. The powerful and in-depth analysis of NGS technology starting from a limited quantity of material and fragmented sequences makes this technology suitable for the study of FFPE tissues.

Here, we investigate the applicability of our custom protocol for isolation of specific histopathological regions of GBM in FFPE tissues, extraction of total RNA, amplification and whole trascriptome analysis (RNA-seq). This method coupled LCM and RNA-seq (LCM-seq) and allows to study the expression profile of each single compartment, thus clarifying its role in the complexity of the tumor.

## Materials and Methods

### Tumor Samples

Three primary human FFPE GBM surgical specimens, diagnosed according to WHO diagnostic criteria (11), were retrieved from the archives of the Tumor Registry of the Anatomy and Pathology Institute of the University of Pisa. Subjects were chosen by the same pathologist, they have same histology, similar conditions and treatments. All cases had a diagnosis of GBM with no previous history of any brain neoplasia and have been diagnosed without R132 IDH1 mutations or R172 IDH2 mutations and 1p/19q co-deletions. Patients underwent maximal tumor resection performed by the same surgeon at the University Hospital of Pisa. All three tumors were located in the right temporal lobe and developed a relapse after 7–8 months from the first surgery. The study was approved by the Ethics Committee of the University Hospital of Pisa and all methods were performed in accordance with approved guidelines. Patient's data and samples have been completely anonymized.

Samples were selected to have six specific histological compartments: central tumor cells (tumor core astrocytes—TC), pseudopalisading cells surrounding the necrotic area (pseudopalisading astrocytes—PTC), infiltrating astrocytes forming perineuronal satellites (perineuronal astrocytes in satellitosis—PS), neurons surrounded by satellite astrocytes (neurons with satellitosis—NS), neo-vessels or microvascular proliferation (tumor blood vessels—TV) and normal blood vessels (NV).

### Laser Capture Microdissection

FFPE tissue blocks were sectioned at 5 μm, mounted on slides covered with polyethylene-naphthalate (PEN)-membrane (Zeiss, Oberkochen, Germany) and left to dry overnight at room temperature. Sections were stained with hematoxylin and eosin (H&E). All steps were performed under RNase-free conditions. Visualization and microdissection was performed with PALM RoboMover Automatic Laser Capture Microdissector (Zeiss). Microdissected areas ranged from 33,700 up to 364,016 μm^2^.

### RNA Isolation

Microdissected areas were directly incubate with 50 μl of lysis buffer PKD (Qiagen, Venlo, Netherlands) and 10 μl of proteinase K solution (Promega, Madison, WI, USA) at 56°C overnight. The day after samples were centrifuged at maximum speed for 10 min. RNA was purified using the Maxwell 16 LEV RNA FFPE Purification Kit (Promega) following manufacturer's instructions.

### SMARTer cDNA Synthesis and Amplification

RNA was reverse transcribed with the SMART (Switching Mechanism at 5' End of RNA Template) technology that allows the efficient incorporation of known sequences at both ends of cDNA during first-strand synthesis, without adapter ligation, using the SMARTer Pico PCR cDNA Synthesis Kit (Clontech Laboratories Mountain View, CA) following manufacturer's instructions. Twenty amplification cycles were required to obtain sufficient library concentration for sequencing. cDNA concentration was determined using the Qubit Fluorometer (Life Technologies, Carlsbad, CA) and the quality was tested using the Agilent 2200 Tapestation (Agilent Technologies, Santa Clara, CA) system.

### NGS Sequencing

For each sample, 50 ng of cDNA were used as input material forge of total RNA will be used as input material for library construction according to Nextera XT DNA Library Preparation Kit (Illumina, San Diego, CA) protocol. Each NGS run included 6 pooled libraries loaded into one NextSeq High Output cartridge (300 Cycles; Illumina). Paired-end sequencing was performed on a NextSeq 500 system (Illumina) with 152 cycles (76 bp PE sequencing) following Encode Project protocol for best RNA Seq data (12). Individual microdissected areas inside each sample are unique to that section and therefore not repeatable through biological replicates. However, the biological triplicate can be considered the one composed of three different samples analyzed for the same areas, as correlation, PCA and differential analyses, evaluate the average value of the three samples.

### Data Analysis

Raw sequencing data were processed with AltAnalyze software (v2.1.0, Cincinnati Children's Hospital, Dr. Nathan Salomonis, Cincinnati, OH, USA) to generate Principal Component Analyses (PCA) and expression clustering profiles of RNA-seq data sets.

Spearman's rank correlation coefficients was used to evaluate our experimental performance by calculating the correlation between RNA-seq raw data experiments of each tumor compartments and between RNA-seq raw data from the BioProject NCBI database (13–18), depending on the cell type, analyzed using the same experimental approach of data analysis. Normal tissue cortex we downloaded from the Gene Expression Omnibus (GEO) GSE102741 dataset (16) and used as normal control.

### Functional Enrichment Analysis

Functional enrichment analysis of the DEGs between tumor compartments and control tissue and between different tumor compartments was performed using FunRich (19) analysis tool. Functional enrichment was carried out for Biological process and Biological pathways.

### Survival Analysis

DEGs from functional enrichment analysis were associated with survival analysis within the glioma microarray dataset (Tumor Glioma French-284-MAS5.0-u133p2) from the R2: Genomics Analysis and Visualization Platform (http://r2.amc.nl). Kaplan-Meier analysis was conducted online, and *p*-values were calculated by the R2 platform user interface with log-rank test. A cutoff method “Kaplan median” provided on the R2 platform was used to separate high and low expression groups of genes.

## Results

### Tumor Histopathology Evaluation

**Figure 1:**
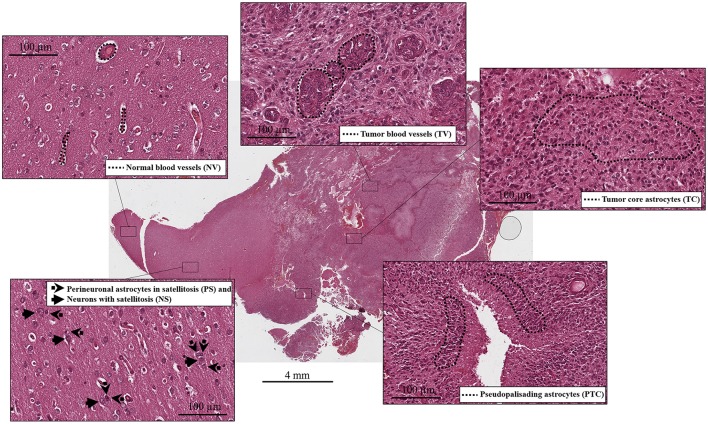
Histological compartments: central tumor cells (tumor core astrocytes—TC), pseudopalisading cells surrounding the necrotic area (pseudopalisading astrocytes—PTC), infiltrating astrocytes forming perineuronal satellites (perineuronal astrocytes in satellitosis—PS), neurons surrounded by satellite astrocytes (neurons with satellitosis—NS), neo-vessels or microvascular proliferation (tumor blood vessels—TV) and normal blood vessels (NV).

Tumor cell purity was around 80% for all samples, with the remaining 20% consisting of hemorrhagic tissue. Tumor core regions represented from 33 up to 67% (Table 1, Figure 1) of the sample. Consistent with glioblastoma, prominent necrosis and microvascular proliferation were visible in each sample (Table 1, Figure 1). A percentage of about 5% of astrocytes migrated into satellites was evident in all three samples (Table 1, Figure 1).

**Table 1:** Histopathologic features of GBM specimens used in this study.

**Sample**	**% Tumor Core**	**% Necrosis**	**% Microvascular Proliferation**	**% Satellitosis**	**% Normal**
8749/2010	52	20	8	5	15
2758/2012	33	40	15	6	6
6475/2007	67	15	7	5	6

*Histopathological evaluation of the three samples in terms of % Tumor Core, tumor core compartment, % Necrosis, necrotic area with pseudopalisading cells, % Microvascular Proliferation, tumor new-vessels, % Satellitosis, astrocytes migrated around neurons, and % Normal (normal vessels, neurons, etc.)*.

### SMARTer Technology Allows an Adequate cDNA Yield Starting From an Area of 30,000 μm^2^ of FFPE Tissue

**Figure 2:**
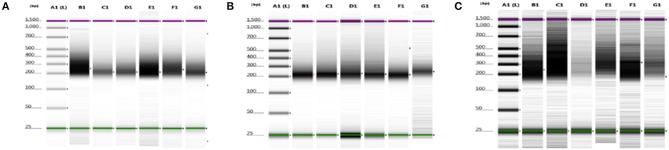
cDNA quality check using gel electrophoresis after SMARTer protocol. **(A–C)** Gel images of 8749/2010, 2758/2012, and 6475/2007 samples, respectively. Purple and green lines are intrinsic upper and lower markers, respectively. **(A–C)** Three independent experiments. Lane A1, ladder; lanes B1–G1 sample compartments (B1, PTC; C1, TC; D1, PS; E1, NS; F1, TV; G1, NV).

We were able to select and isolate cellular compartment areas ranging from 33,700 up to 364,016 μm^2^. An average of 23.2 ± 4.7 ng/μl cDNA was obtained from all portions of the three samples. Total cDNA yield and integrity were evaluated as suitable to start library construction (Figure 2).

Averages of total areas of collected cells and cDNA yields, after SMARTer protocol, between the 3 samples, for each compartment, are listed in Table 2.

**Table 2:** cDNA yield after SMARTer protocol.

***N***	**Compartment**	**Area (μm^**2**^)**	**cDNA (ng)**
3	TC	≈364016	≈17.4
3	PTC	≈103000	≈ 29.0
3	PS	≈33700	≈27.0
3	NS	≈39000	≈25.0
3	TV	≈78000	≈22.8
3	NV	≈76800	≈18.0

*Averages of total areas (μm^2^) of collected cells and cDNA yields (ng) after SMARTer protocol, between the 3 samples (N), for each compartment*.

### LCM-Seq Is a Reproducible and Sensitive Approach for Downstream Molecular Analysis

**Figure 3:**
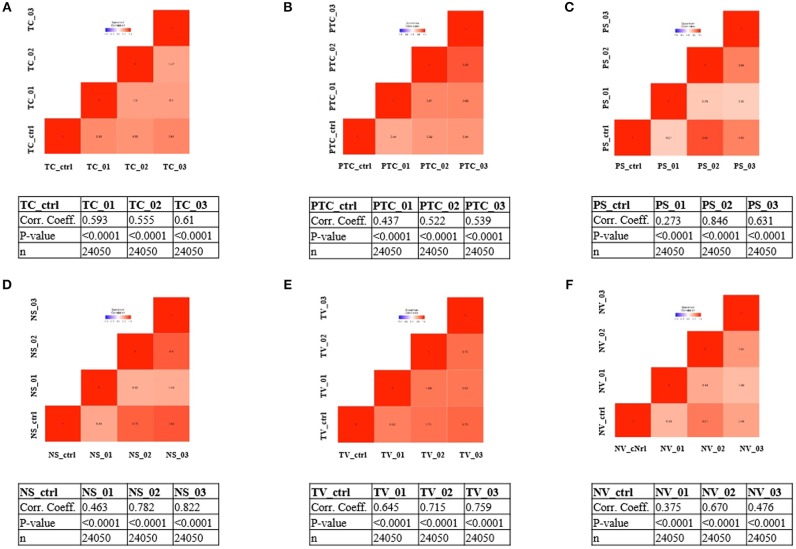
Correlation matrix showing Spearman's correlation coefficients of each experimental compartment RNA-seq raw data and experiments from the BioProject NCBI database as controls. **(A)** tumor core astrocytes—TC; **(B)** pseudopalisading astrocytes—PTC; **(C)** perineuronal astrocytes in satellitosis—PS; **(D)** neurons with satellitosis—NS; **(E)** tumor blood vessels—TV; **(F)** normal blood vessels—NV.

To address whether the expression pattern of RNA obtained from LCM and linear amplification (Supplementary Table 1) accurately reflects that of the corresponding cell compartment, we computed Spearman's correlation coefficients using RNA-seq raw data experiments from the BioProject NCBI database as controls (Table 3). The correlation score shows an average coefficient up to 0.65 for tumor core, microvascular proliferation and neurons (*p* < 0.001), while pseudopalisading cells and normal vessels show an average coefficient of 0.5 (*p* < 0.05), (Figure 3, Table 3). Moreover, to investigate the reliability and the integrity of our NGS transcription data results for each compartment, we evaluated the expression of genes considered as housekeeping candidates in human normal brain tissue, glioblastoma and endothelial cells (20–22) (RPL13A, RPL4, CYC1, EIF4A2).

**Table 3:** Transcription data results and Spearman correlation score.

**Compartment**	**Mapped reads**	**RPL13A**	**RPL4**	**CYC1**	**EIF4A2**	**RNA-seq Ref**	**Spearman Score**
TC	1895370.67	3.36	89.98	16.36	155.51	13	0.586^***^
PTC	1471923.5	133.13	42.21	26.74	101.90	14	0.499^***^
PS	2361735.25	253.36	103.43	20.30	40.11	15	0.583^***^
NS	2357694.75	84.78	65.00	19.30	1150.21	16	0.689^***^
TV	5857616.25	211.81	135.09	60.20	95.06	17	0.706^***^
NV	3733412.5	231.98	196.95	9.83	111.91	18	0.507^***^

*Mapped reads, number of mapped alignments to the human genome Crh38; TBP, RPL13A, RPL4, CYC1, EIF4A2, number of fragments mapping to the genome per kilobase of transcript per million (FPKM) reads sequenced; RNA-seq Ref, references of the BioProject NCBI database data; Spearman Score, Spearman correlation test calculated as average of three experiments compared to RNA Seq data available from the BioProject NCBI database. Data are considered statistically significant when p < 0.05 and represented as*:

*** *p < 0.001*.

**Figure 4:**
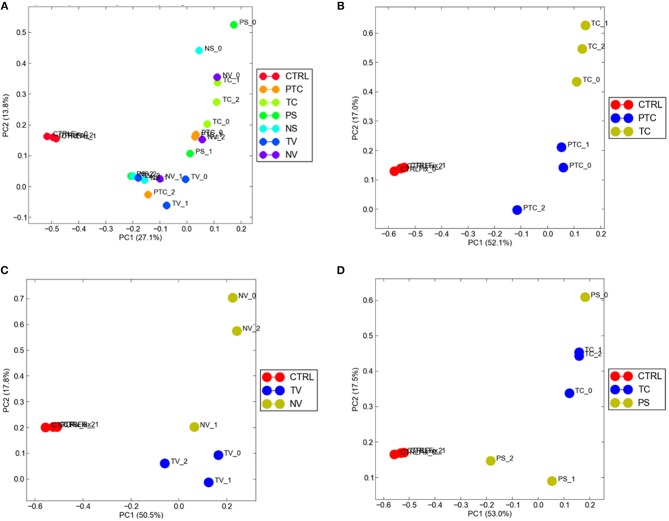
Distribution of normal tissues and cancer subtypes in the principal component space. **(A)** control tissue and the tumor samples are represented in two different areas of the 2D plots in the first dimension (PC1); gene expression diversity was captured and segregated in three different areas by the second dimension (PC2) when compared TC and PTC **(B)**, TC and PS **(C)**, TV and NV **(D)**.

To visualize gene expression differences between each cellular subgroup and control tissue, we performed principal-component analysis (PCA) using all tumor compartments compared to control tissue RNA-seq raw data experiments from the Gene Expression Omnibus (GEO) GSE102741 dataset (16). PCA showed that control tissue and the tumor samples groups always segregated in two different areas of the 2D plots in the first dimension (PC1), (Figure 4A). 8585 mRNA genes were Differentially Expressed (DEGs) when considering a significance threshold of *p* < 0.05.

When comparing two different compartments to each other and to control, gene expression diversity was captured and segregated in three different areas by the second dimension (PC2). We observed gene expression differences comparing TC and PTC (Figure 4B), TC and PS (Figure 4C), and between TV and NV (Figure 4D).

### Functional Enrichment Analysis of DEGs Based on RNA-Seq Data

#### GBM and Control Tissues

To identify possible pathways that are significantly associated with the tumor, functional enrichment analysis was first computed on 8,585 differentially regulated genes (Supplementary Table 1), that contribute to the largest variation in PC1-axis (Figure 2A). Functional enrichment investigation shows biological processes and pathways deregulation in tumor compartments compared to control tissue, when considered DEGs with *p* < 0.05 (Table 4). The majority of DEGs belonged to the signal transduction/cell communication biological processes, including the tyrosine kinase-type cell surface receptor 4 (HER4), Filamin B (FLNB), Integrins (ITGA2, ITGA4,ITGA8, ITGA9, ITGB7, ITGBL1) and the proto-oncogene tyrosine-protein kinase SRC. Among the DEGs involved in transmembrane transport of small molecules we found aquaporins genes (AQP6, AQP7, AQP9, AQP10, AQP11). The most representative DEGs involved in cell growth and/or maintenance take part to the remodeling and homeostasis of the extracellular matrix (ECM). The expression of metalloproteinases and their inhibitors (MMP15, MMP16, MMP17, MMP24, TIMP1), laminins (LAMA2, LAMA3), collagen gene family members (COL4A2, COL4A3, COL4A3BP, COL4A5, COL6A1, COL9A2, COL10A1, COL11A1, COL11A2, COL12A1, COL17A1, COL18A1, COL19A1, COL21A1, COL24A1, COL25A1, COL26A1, COL27A1, COL28A1, COLGALT2) and tight junction components and adaptors (CLDN1, CLDN3, CLDN4, CLDN6, CLDN9, CLDN10, CLDN11, CLDN16, CLDN20, CLDN24, TJP1, TJP2, TJP3) was found altered in the tumor compartments compared to normal control tissue.

**Table 4:** Top 5 biological processes and relative pathway annotations for DEGs between all tumor compartments and control tissue.

**Biological process**	**% of genes**	**Biological pathway**	**No of genes**
Signal transduction/Cell communication	23	ErbB receptor signaling network Integrin family cell surface interactions PAR1-mediated thrombin signaling events VEGF and VEGFR signaling network PDGF receptor signaling network	188 185 183 182 181
Metabolism	10	Metabolism of lipids and lipoproteins Metabolism of amino acids and derivatives Biological oxidations The citric acid (TCA) cycle and respiratory electron transport Post-translational protein modification	56 41 36 28 21
Transport	8	Transmembrane transport of small molecules Potassium channels Transport of glucose and other sugars, bile salts and organic acids, metal ions and amine compounds Arf6 trafficking events Ion transport by P-type ATPases	109 39 27 15 13
Cell growth and/or maintenance	6	Class I PI3K signaling events mediated by Akt Mitotic G2-G2/M phases DNA Replication Regulation of CDC42 activity Apoptosis	18 14 13 11 7
Immune response	2	Nectin adhesion pathway Beta1 integrin cell surface interactions Endothelins GMCSF-mediated signaling events Insulin/IGF1 Pathways	19 19 19 18 18

*% of genes, percentage of DEGs involved in a specific process; Biological pathway, main biological pathways in which selected DEGs are involved, in order of number of DEGs; No of genes, number of DEGs involved in a specific pathway*.

#### Pseudopalisading Areas and Tumor Core

**Figure 5:**
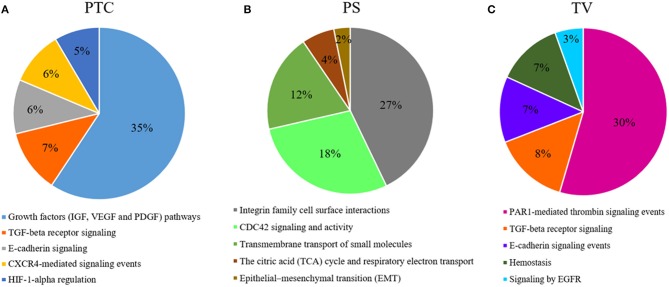
Functional enrichment analysis of DEGs. Top 5 biological processes up-regulated with percentage of DEGs involved in a specific pathway in **(A)**, PTC to pseudopalisading astrocytes and, **(B)** PS perineuronal astrocytes comparing to TC tumor core astrocytes and, **(C)** TV tumor blood vessels comparing to normal blood vessels.

DEGs analysis showed a total of 249 genes (Supplementary Table 1), of which 15 down-regulated and 234 up-regulated in pseudopalisading areas, when compared to tumor core areas. Functional enrichment analysis highlighted up-regulated DEGs of pseudopalisading compartments in TOP 5 biological pathways, according to DEGs number (Figure 5A). In particular, we found an overexpression of proangiogenic genes and pathways and genes involved in cell migration in PTC cells, such as Angiopoietin 2 (ANGPT2), Urokinase Plasminogen Activator Receptor (PLAUR), Growth Differentiation Factor 15 (GDF15), Matrix Gla Protein (MGP), Proto-oncogene serine/threonine-protein kinase Pim-1 (PIM1), Cullin 1 (CUL1), and Mitogen-activated protein kinases—MAPK (MAP3K6 and MAPK7).

#### Astrocytes in Satellitosis and Tumor Core

Comparing astrocytes in satellitosis to astrocytes in the tumor core, we found 358 DEGs (Supplementary Table 1). Two hundred and fifty six DEGs were specifically up-regulated in neoplastic astrocytes in satellitosis. We explored individual pathways from functional enrichment analysis, finding the TOP 5 biological pathways (Figure 5B) up-regulated in astrocytes in satellitosis. PS compartment overexpresses specific metalloproteinases (MMP9 and MMP28) and aquaporins (AQP1 and AQP4). Furthermore, PS cells overexpressed BRCA1 and Secreted Protein Acidic and Cysteine Rich like protein 1 (SPARCL1).

#### Tumor and Normal Vessels

Functional enrichment analysis computed on 1,049 up-regulated genes in tumor vessels compared to normal vessels, out of 1,157 total DEGs (Supplementary Table 1), shows (Figure 5C) the TOP 5 biological pathways in which DEGs are involved. Endothelial cells of tumor neo-vessels, over-expressed a string of DEGs correlated with the signaling pathways that govern tumor angiogenesis, including the vascular endothelial growth factor receptor 3 (VEGFR3 or FLT4), HIF-1A, ANGPT2, Metalloproteinases (MMP11 and MMP15) and Integrins (ITGA2, ITGAE, and ITGB1BP1).

### Association Between DEGs Expression and Prognostic Survival

The association between DEGs from functional enrichment analysis and survival outcome is shown in Table 5 (Kaplan Meier survival curves are available in Supplementary Figures 1–4). We selected the top 5 significant associations within DEGs between GBM and Control Tissues and the top 5 significant associations within overexpressed DEGs of PTC cells vs. CT cells, PS cells vs. CT cells and TV vs. NV endothelial cells.

**Table 5:** Association between DEGs from functional enrichment analysis and survival outcome.

	**DEG**	**Overexpression**	**Worse prognosis**	***p*-value**
**GBM AND CONTROL TISSUES**				
1	TIMP1	Tumor	High expression	1.3e^−13^
2	COL6A1	Tumor	High expression	6.8e^−10^
3	COL4A2	Tumor	High expression	5.3e^−9^
4	MMP16	Control	Low expression	2.8e^−7^
5	AQP11	Control	Low expression	3.4e^−6^
**PSEUDOPALISADING AREAS AND TUMOR CORE**				
1	MAP3K6	Pseudopalisading areas	High expression	1.3e^−14^
2	PLAUR	Pseudopalisading areas	High expression	5.8e^−13^
3	GDF15	Pseudopalisading areas	High expression	1.5e^−10^
4	CUL1	Pseudopalisading areas	High expression	1.2e^−4^
5	MGP	Pseudopalisading areas	High expression	7.8e^−4^
**ASTROCYTES IN SATELLITOSIS AND TUMOR CORE**				
1	AQP1	Astrocytes in satellitosis	High expression	2.8e^−10^
2	MMP9	Astrocytes in satellitosis	High expression	7.2e^−7^
3	BRCA1	Astrocytes in satellitosis	High expression	2.6e^−6^
4	AQP4	Astrocytes in satellitosis	High expression	5.3e^−5^
5	SPARCL1	Astrocytes in satellitosis	High expression	0.039
**TUMOR AND NORMAL VESSELS**				
1	ITGA2	Tumor vessels	High expression	2.0e^−12^
2	ITGB1BP1	Tumor vessels	High expression	2.2e^−8^
3	HIF1A	Tumor vessels	High expression	7.7e^−6^
4	MMP11	Tumor vessels	High expression	5.6e^−5^
5	FLT4	Tumor vessels	High expression	0.045

*DEG, DEGs from transcriptional status of GBM compartments vs. control tissue, PTC cells vs. CT cells, PS cells vs. CT cells and TV vs. NV endothelial cells; Overexpression, DEG overexpression in a specific tumor compartment or tissue; Worse prognosis, DEG overexpression or downregulation associated with a worse prognosis (short overall survival); p-value, significance of the log-rank test*.

## Discussion

GBM is a devastating brain tumor disease for which no effective therapies are available (23). Large-scale genetic investigations have identified several mutations in key genes in GBM and TCGA consortium have provide molecular subtypes classification systems to specifically stratify GBM patients (24, 25). Nonetheless, the causes of GBM recurrence and drug resistance are still unknown. Although astrocytes are the most affected cells in GBM, other cell types such as microglia, oligodendrocytes, neurons and pericytes in different histological compartment may contribute to progression and to relapse of the disease (26). Moreover, GBM is also compartmentalized in anatomically distinct regions, referred to as morphologically and functionally distinct tumor niches (27).

Previous studies have interrogated tumor heterogeneity by analyzing the transcriptome profile of fresh/frozen primary tumor tissues (28, 29) and tumor single-cells (30–32). However, these studies still lack a systematic understanding of the molecular heterogeneity of the tumor in relation to anatomic heterogeneity. In this regard, further studies have assigned genomic alterations and gene expression profiles to specific anatomical features of glioblastoma. These investigations explored regional intratumoral differences in tumor periphery and core regions (14, 33), such as one has also considered the necrotic zone (34), while the most complete (35, 36), from which the Ivy Glioblastoma Atlas originated, analyzed 5 different tumor regions (leading edge, infiltrating tumor, cellular tumor, pseudopalisading cells around necrosis, and microvascular proliferation). The use of fresh/frozen samples, compared to FFPE tissues, certainly increases the quality of both extracted RNA and gene expression data, but does not allow to discriminate tumor compartments edges and structures, at the single cells level.

To characterize whole transcriptome modifications related to specific compartments, we conducted a LCM-seq study of 6 different tumor cell subpopulations in 3 human FFPE GBM samples: tumor core astrocytes—TC, pseudopalisading astrocytes—PTC, perineuronal astrocytes in satellitosis—PS, neurons with satellitosis—NS, tumor blood vessels—TV and normal blood vessels—NV. We first validated our data comparing LCM-seq expression values of each compartment to gene expression levels of RNA-Seq raw data from the Bio Project databases of NCBI (13–18), by applying Spearman correlation test (37) and finding good concordance in each dissected tumor compartment.

We performed, next, a principal component analysis (PCA), demonstrating that samples originating from each experimental group were clustered and indicating that each tumor compartment exhibit distinct gene expression profile. RNA-Seq data of normal human cerebral cortex tissue (16) was used as control to find differential expressed genes compared to all tumor groups. Our results shown a gene expression alteration of the principal genes involved in tumorigenesis and cancer progression mostly of them linked to signal pathways in glioblastoma. The majority of DEGs belonged to the signal transduction/cell communication biological processes. These DEGs have been already described in literature in association with GBM patient survival (38) and GBM tumor progression (39–43). An alteration of metabolism processes has also been found altered in our tumor samples, above all, the metabolism of lipids and amino acids, known to be altered in GBM (44–46). Among the DEGs involved in transmembrane transport of small molecules we found aquaporins genes. Several studies have shown the involvement of aquaporins in many aspects of brain pathogenesis, such as promotion of tumor cells motility and invasion, as well as formation of edema and improvement of tumor cells glycolytic metabolism (47). The most representative DEGs involved in cell growth and/or maintenance take part to the remodeling and homeostasis of the extracellular matrix (ECM). In GBM progression, the role of the ECM in cell migration and invasion and its correlation with patient survival has already been fully established (48–59).

RNA-Seq data of the single selected regions provide a snapshot of transcriptomic events that identify the current state of the tumor, characterized by the up and down-regulation of several genes. Although our data do not provide evidence on early molecular events, transcriptomic results seem to support that certain molecular events are region specific and each process is strictly dependent on others. We, therefore, tried to point out specific ongoing processes in each individual compartment, highlighting which are the up- or down-regulated genes of the specific microenvironment and not of the whole tumor. We have compared the transcriptional status of PTC cells vs. CT cells, PS cells vs. CT cells and TV vs. NV endothelial cells.

Our results of DEGs analysis in pseudopalisading cells compared to tumor core showed a considerable (35% of DEGs) up-regulation in genes involved in growth factors (IGF, VEGF, and PDGF) signaling pathways. Moreover, in PTC cells, we found an overexpression of genes belonged to TGF-beta receptor signaling, E-cadherin signaling, CXCR4-mediated signaling events and Hypoxic and oxygen homeostasis regulation of HIF-1-alpha. Pseudopalisades are described as waves of tumor and hypoxic cells that actively migrate away from an area of central hypoxia. These cells are known to overexpress the inducible hypoxia factor-1 (HIF-1) and other transcripts that suggest a response to a hypoxic microenvironment, such as those related to glycolysis, angiogenesis, and cell cycle control (7, 60). In particular, we found an overexpression of proangiogenic genes and pathways and genes involved in cell migration in PTC cells, known to promote cell survival and infiltrative growth, migration, angiogenesis and resistance to cancer-targeted therapies in GBM (61–67). Moreover, MAPKs participate in the regulation of vascular endothelial growth factor (VEGF) expression (68) and lead to the elevated level of HIF-1 protein which act as proangiogenic factor promoting cancer angiogenesis (69).

To explain the spread of neoplastic astrocytes to normal brain parenchyma, we have collected astrocytes around neurons in a specific histological configuration, named perineuronal satellitosis. This phenomenon is not even detectable through sophisticated surgical approaches and neuro-imaging acquisition but only appreciable at the histological level. Evidence suggest that astrocytes can be implicated in tumor propagation and in infiltration. Besides moving over long distances along myelinated fiber tracts and blood vessels, astrocytes can also cluster around neuronal soma (27, 70). Isolated astrocytes in neuronal satellitosis displayed a high activation of the Integrin family cell surface interactions pathways, responsible for the interaction of endothelial and tumor cells with the ECM (71). Other pathways represented by various DEGs overexpressed by astrocytes in satellitosis are the cell division control protein 42 homolog (CDC42) signaling and activity pathways. Previous studies have already demonstrate a correlation between CDC42 activation and increased aggressiveness and invasiveness of malignant gliomas (72). The other most representative pathways of this compartment are the transmembrane transport of small molecules, the citric acid (TCA) cycle and respiratory electron transport and epithelial-mesenchymal transition (EMT). PS compartment overexpresses specific metalloproteinases and aquaporins, crucial in cell migration as already mentioned above. Furthermore, PS cells overexpressed BRCA1 and SPARCL1, already known in GBM to promote tumor cell viability, migration and invasion and to correlate with patients prognosis (73, 74).

The presence of microvascular proliferation is one of the most important morphologic features of glioblastoma (75). Several mechanisms have been involved in blood vessels formation of GBM, such as germination of capillaries from pre-existing blood vessels through endothelial proliferation and tumor cells release of angiogenic factors (76). Blocking VEGF/VEGFR signaling, to reduce and trim the growth of tumor vessels, emerged as the first promising treatment strategy in GBM patients. However, to date, the anti-VEGF therapy has helped only a small subset of GBM patients, and those patients demonstrated only transient improvements without achieving overall survival benefits (27). Most of the DEGs overexpressed in the endothelial cells of the tumor neo-vessels, compared to those of the normal vessels, are grouped by the functional enrichment analysis under the PAR1-mediated thrombin signaling events pathway. PAR-1 plays an important role in angiogenesis and its expression is also directly associated with increased VEGF levels (77). FLT4 DEG is not expressed in endothelium of normal brain, in physiological adult tissues, but its mRNA was found only in high-grade gliomas and its expression has been correlated with tumor grade (78). Other angiogenetic key factors, such as HIF-1A, ANGPT2, Metalloproteinases, and Integrins were found over-expressed in the endothelial cells of microvascular proliferation.

## Conclusions

In this study, we provide a feasible and reliable method for isolating pure GBM cell populations from different histological compartments, with LCM approach, minimizing cross-contamination. Our results demonstrate the suitability of LCM coupled to deep transcriptome sequencing for capturing molecular changes in different GBM compartments and investigating the tumor heterogeneity. Our results are promising and suggest that LCM-seq is a sensitive technology that may be used to study FFPE specimens in both prospective and retrospective archive-based studies. Transcriptome profile of neurons with satellitosis (NS) will be used in future studies in the comparison to neurons microdissected from healthy marginal areas. This will allow a better characterization also of the interactions occurring between the astrocytes surrounding the neuron and the neuron itself.

## Data Availability

This manuscript contains previously unpublished data. The name of the repository and accession number are not available.

## Ethics Statement

The study was approved by the Ethics Committee of the University Hospital of Pisa and all methods were performed in accordance with approved guidelines. Patient's data and samples have been completely anonymized.

## Author Contributions

AN, SF, CM, and PC conceived the idea. FP provided patient data and material. PC, MM, and FL contributed to the sample-preparations. PC, MM, and FL carried out the laboratory analyses. VO and MM performed laser capture microdissection. PA performed the statistics. SF and PC analyzed and interpreted the data. CM was involved in the planning and supervising. SF and PC wrote the manuscript and designed the figures; contributed equally to this work. All authors read and approved the final manuscript.

### Conflict of Interest Statement

The authors declare that the research was conducted in the absence of any commercial or financial relationships that could be construed as a potential conflict of interest.
