# Supplementary material for: Laser Capture Microdissection and RNA-Seq Analysis: High Sensitivity Approaches to Explain Histopathological Heterogeneity in Human Glioblastoma FFPE Archived Tissues
Source: Front Oncol. 2019 Jun 7;9:482. doi: 10.3389/fonc.2019.00482 (PMC6568189; doi:10.3389/fonc.2019.00482)

Supplementary Figure S1. The correlation of tumor VS control tissues DEGs levels with the overall survival of glioma patients. Association between high and low expression patterns of (**A**) TIMP1, (**B**) COL6A1, (**C**) COL4A2, (**D**) MMP16 and (**E**) AQP11 and overall survival time based on the R2 database, presented by Kaplan-Meier plots.

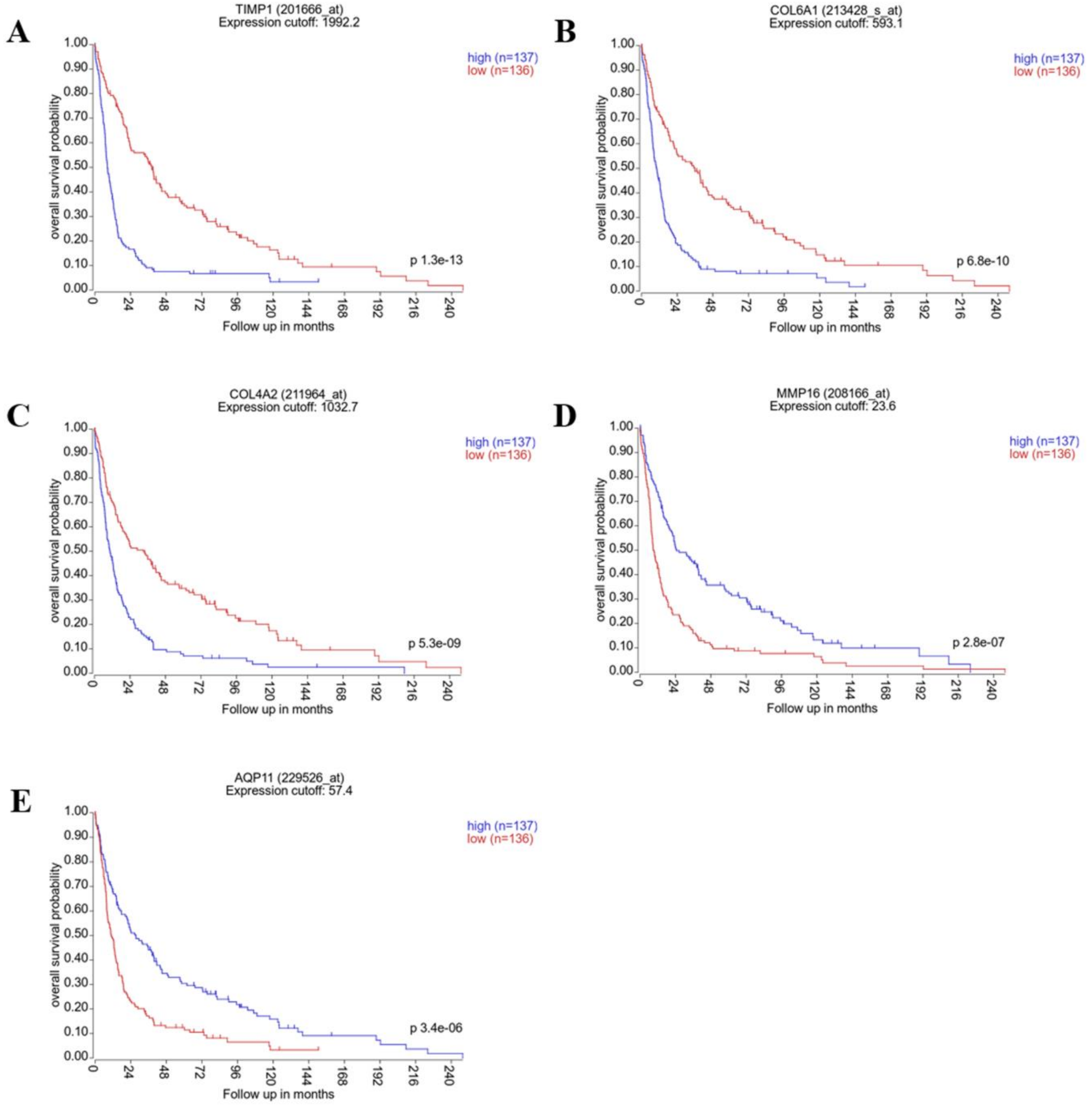

Supplementary Figure S2. The correlation of pseudopalisading areas DEGs levels with the overall survival of glioma patients. Association between high and low expression patterns of (**A**) MAP3K6, (**B**) PLAUR, (**C**) GDF15, (**D**) ANGPT2 and (**E**) MGP and overall survival time based on the R2 database, presented by Kaplan-Meier plots.

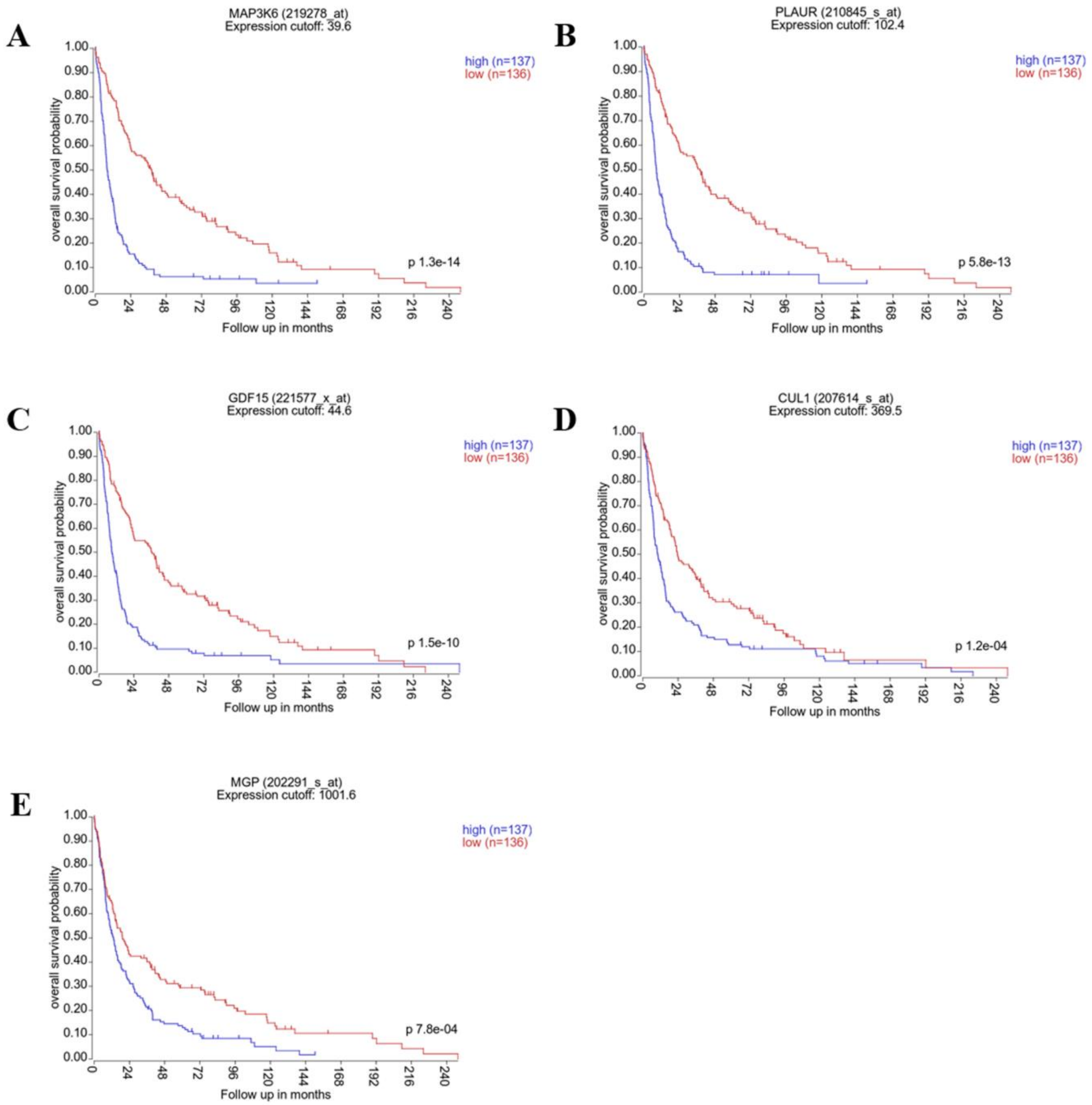

Supplementary Figure S3. The correlation of astrocytes in satellitosis DEGs levels with the overall survival of glioma patients. Association between high and low expression patterns of (**A**) AQP1, (**B**) MMP9, (**C**) BRCA1, (**D**) AQP4 and (**E**) SPARCL1 and overall survival time based on the R2 database, presented by Kaplan-Meier plots.

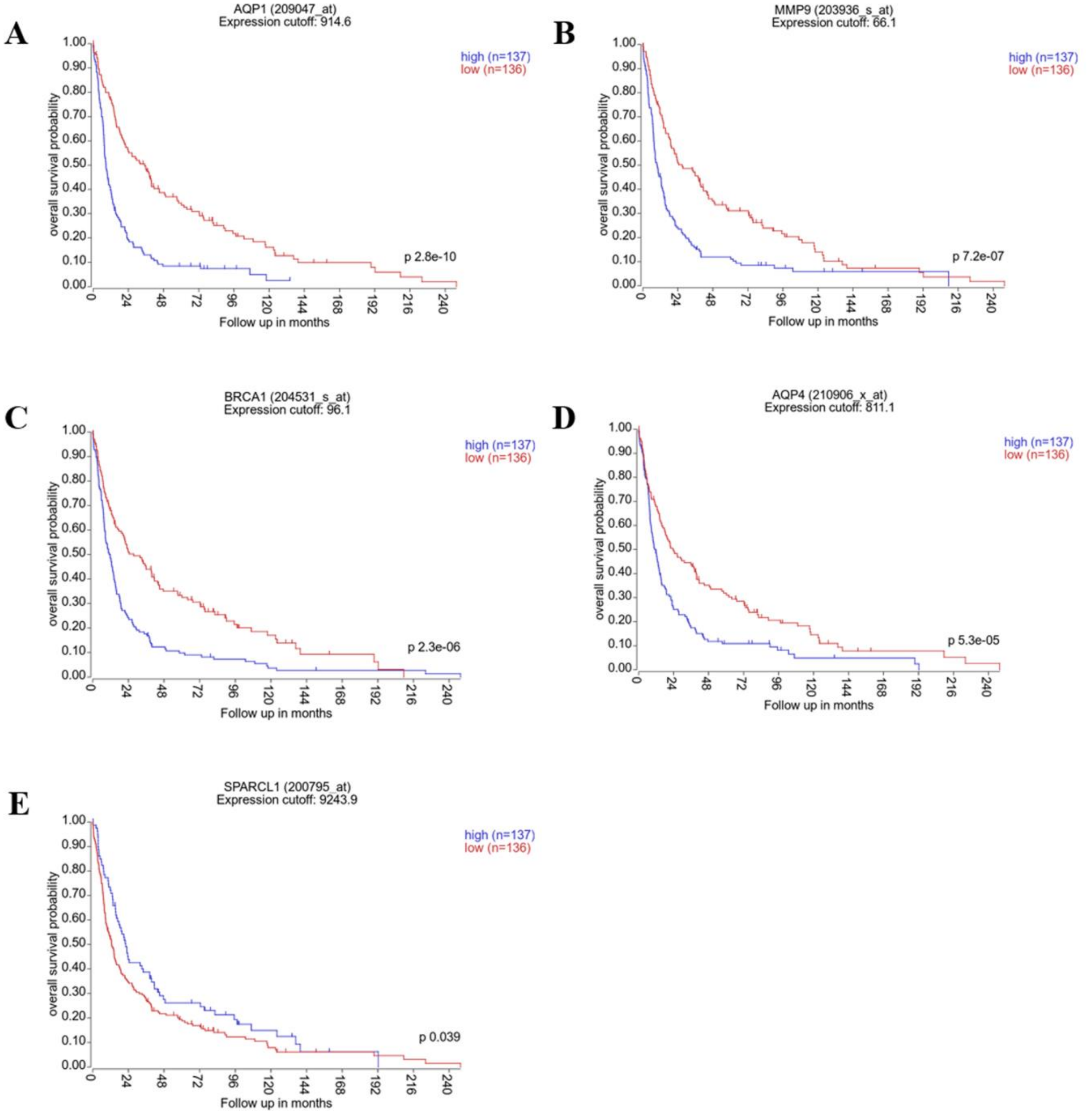

Supplementary Figure S4. The correlation of tumor vessels DEGs levels with the overall survival of glioma patients. Association between high and low expression patterns of (**A**) ITGA2, (**B**) ITGB1BP1, (**C**) HIF1A, (**D**) MMP11 and (**E**) FLT4 and overall survival time based on the R2 database, presented by Kaplan-Meier plots.

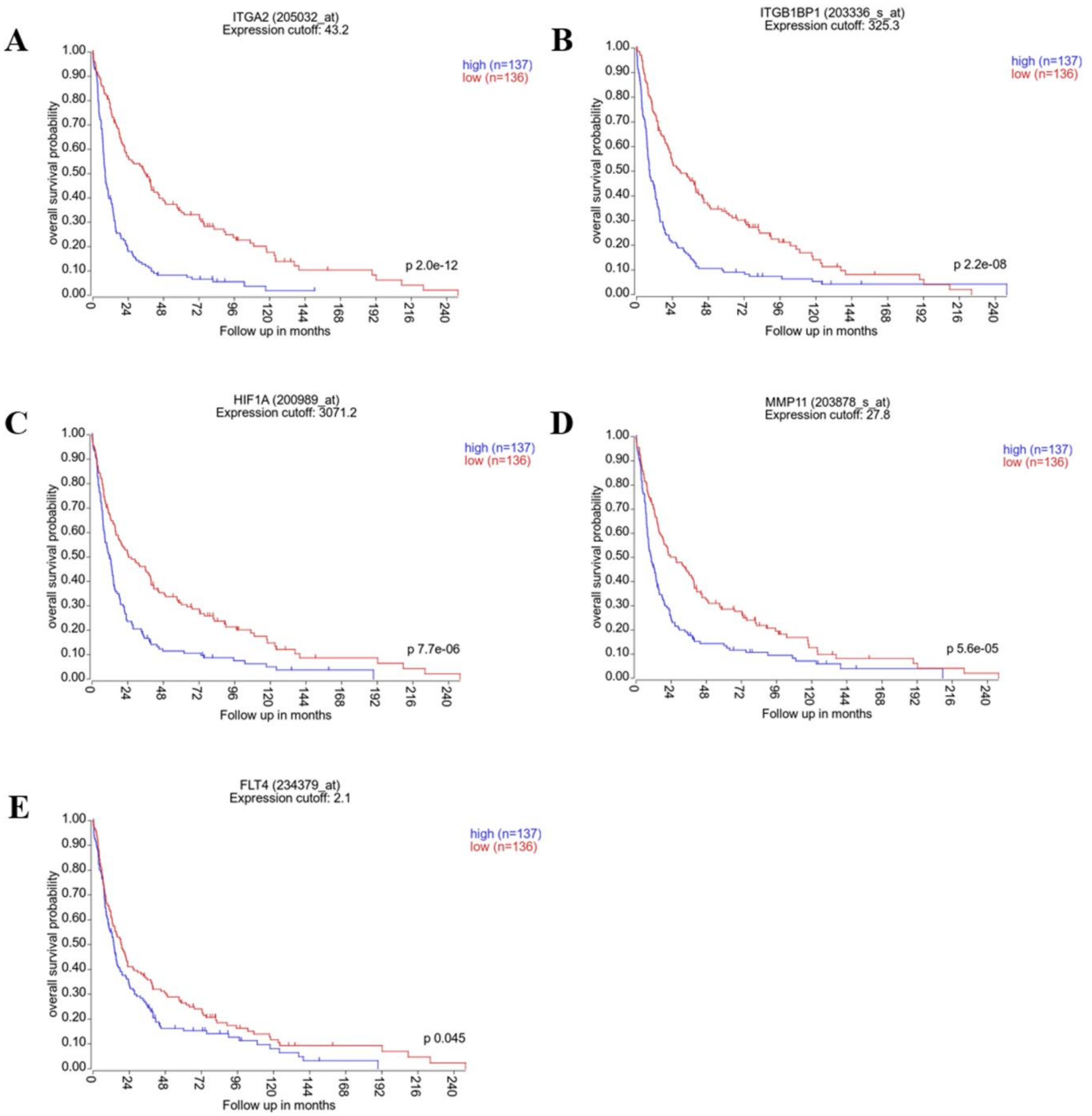

Supplement: Supplementary file 1 [file Data_Sheet_1.PDF]
